# ATM Deficiency Accelerates DNA Damage, Telomere Erosion, and Premature T Cell Aging in HIV-Infected Individuals on Antiretroviral Therapy

**DOI:** 10.3389/fimmu.2019.02531

**Published:** 2019-11-05

**Authors:** Juan Zhao, Lam Ngoc Thao Nguyen, Lam Nhat Nguyen, Xindi Dang, Dechao Cao, Sushant Khanal, Madison Schank, Bal Krishna Chand Thakuri, Stella C. Ogbu, Zheng D. Morrison, Xiao Y. Wu, Zhengke Li, Yue Zou, Mohamed El Gazzar, Shunbin Ning, Ling Wang, Jonathan P. Moorman, Zhi Q. Yao

**Affiliations:** ^1^Center of Excellence in Inflammation, Infectious Disease and Immunity, James H. Quillen College of Medicine, East Tennessee State University, Johnson City, TN, United States; ^2^Division of Infectious, Inflammatory and Immunologic Diseases, Department of Internal Medicine, Quillen College of Medicine, East Tennessee State University, Johnson City, TN, United States; ^3^Hepatitis (HCV/HBV/HIV) Program, James H. Quillen VA Medical Center, Department of Veterans Affairs, Johnson City, TN, United States

**Keywords:** ATM, apoptosis, DNA damage repair, immune aging, HIV, T cell homeostasis

## Abstract

HIV infection leads to a phenomenon of inflammaging, in which chronic inflammation induces an immune aged phenotype, even in individuals on combined antiretroviral therapy (cART) with undetectable viremia. In this study, we investigated T cell homeostasis and telomeric DNA damage and repair machineries in cART-controlled HIV patients at risk for inflammaging. We found a significant depletion of CD4 T cells, which was inversely correlated with the cell apoptosis in virus-suppressed HIV subjects compared to age-matched healthy subjects (HS). In addition, HIV CD4 T cells were prone to DNA damage that extended to chromosome ends—telomeres, leading to accelerated telomere erosion—a hallmark of cell senescence. Mechanistically, the DNA double-strand break (DSB) sensors MRE11, RAD50, and NBS1 (MRN complex) remained intact, but both expression and activity of the DNA damage checkpoint kinase ataxia-telangiectasia mutated (ATM) and its downstream checkpoint kinase 2 (CHK2) were significantly suppressed in HIV CD4 T cells. Consistently, ATM/CHK2 activation, DNA repair, and cellular functions were also impaired in healthy CD4 T cells following ATM knockdown or exposure to the ATM inhibitor KU60019 *in vitro*, recapitulating the biological effects observed in HIV-derived CD4 T cells *in vivo*. Importantly, ectopic expression of ATM was essential and sufficient to reduce the DNA damage, apoptosis, and cellular dysfunction in HIV-derived CD4 T cells. These results demonstrate that failure of DSB repair due to ATM deficiency leads to increased DNA damage and renders CD4 T cells prone to senescence and apoptotic death, contributing to CD4 T cell depletion or dysfunction in cART-controlled, latent HIV infection.

## Introduction

Aging is associated with, and may be caused by, low-grade inflammation—a phenomenon known as inflammaging. Inflammaging is a major driver of age-related phenomena such as increased susceptibility to infections, reduced responses to vaccines, and increased incidence of cancers, cardiovascular diseases, and neurodegeneration, all of which contribute to comorbidity and mortality in the elderly (1–3). In the human immune system, CD4 T cells are long-lived, and thus are exposed to extensive genomic insults and replicative pressure, making them vulnerable to aging-associated abnormalities. Notably, the progressive loss of T cell proliferative capacity during the aging process directly correlates with the gradual shortening of telomeres—a hallmark of cell senescence. Therefore, telomere length has been deemed as a biological clock controlling cell aging, survival, and function, whereas telomere attrition has been considered a faithful readout of inflammaging (1–3).

Growing evidence suggests that this aging phenotype is recapitulated in chronic viral infections, particularly in human immunodeficiency virus (HIV) infection, which is characterized by disrupted CD4 T cell homeostasis and accelerated premature immune aging (4). Many of the alterations that affect the immune system in HIV-infected individuals are reminiscent of the progression of inflammaging in the elderly (5, 6). Currently, combined antiretroviral therapy (cART) does not always result in complete CD4 T cell recovery (7–10). Despite successful control of HIV replication by cART, residual inflammaging persists, leading to significant immune dysfunction. Even with reasonable CD4 T cell counts, these cART-controlled HIV patients often experience profound inflammaging, characterized by extremely short telomeres, low IL-2 production, poor cellular proliferation, and blunted vaccine responses (5–16). This exposes the immune system to unique challenges that lead to T cell exhaustion and senescence, similar to that observed in the elderly. These alterations accelerate the decline of CD4 T cell competence and thus define the overall immune dysfunction during HIV latency. Therefore, cART-controlled, latent HIV infection can be deemed as an excellent model of inflammaging in humans; as such, it is very important to elucidate the mechanisms underlying T cell senescence, telomere erosion, and inflammaging in latent HIV infection.

Because telomeric DNA damage can cause telomere erosion, leading to premature cell aging and/or apoptosis (17, 18), we hypothesized that progressive CD4 T cell loss and failure of immune recovery during latent HIV infection could be, at least in part, due to a deficiency in the DNA repair mechanism that alters telomere integrity. Indeed, we have previously shown that patients with chronic hepatitis C virus (HCV) infection exhibit premature T cell aging, evidenced by overexpressed aging markers and particularly, shortened telomeres—indicative of excessive proliferative turnover or inadequate telomeric maintenance (19, 20). Notably, in normal primary T cells, telomeres undergo shortening at a rate of 50–100 base pairs (bp) per cell division, and this predictable loss of telomeric DNA with cell replication allows telomeres to serve as a molecular clock that controls the replicative capacity of human T cells before entering cell cycle arrest, senescence, or apoptosis (21, 22). However, telomere loss can increase up to 250 bp per cell division during viral infection, and in compensating for this, cell cycle arrest occurs when progressive telomere loss reaches a critical point—known as senescence (a quiescent, non-replicative state with a decline in functional activities) (21, 22). Thus far, the precise mechanisms controlling telomeric DNA damage, T cell homeostasis, inflammaging, and immune senescence in latent HIV infection remain unclear.

In this study, we investigated the molecular mechanisms that perturb T cell homeostasis and lead to inflammaging using a model of HIV-infected individuals on cART with undetectable viremia—the state in which the majority of HIV patients currently exist. We demonstrate that deficiency of ataxia-telangiectasia mutated (ATM) kinase promotes DNA damage, telomere erosion, and T cell senescence and apoptosis in latent HIV infection. Specifically, we show that cART-controlled HIV infection is characterized by telomeric DNA damage that remains unrepaired due to ATM deficiency, leading to telomere attrition and CD4 T cell senescence or apoptosis, providing a novel mechanism controling T cell homeostasis and premature immune aging. Thus, restoring impaired ATM machinery may provide a new strategy to improve T cell survival and functions in cART-controlled HIV infection.

## Results

### CD4 T Cell Homeostasis and Apoptotic Susceptibility in cART-Controlled, Latent HIV Infection

**Figure 1:**
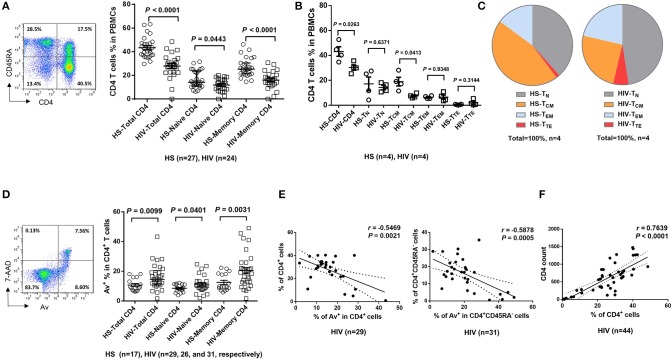
T cell homeostasis and apoptosis in HIV-infected patients vs. age-matched HS. **(A)** PBMCs were isolated from 24 HIV-infected patients and 27 HS and analyzed for T cell homeostasis by flow cytometry. Representative pseudocolor plots and summary data for total CD4^+^, CD45RA^+^CD4^+^ (naïve), and CD45RA^−^CD4^+^ (memory) T cell frequencies are shown. Each symbol represents an individual subject. The mean ± SEM or median ± interquartile range and *p*-value are shown. **(B)** Flow cytometry was used to analyze the the frequencies of total CD4^+^, CD4^+^CD45RA^+^CCR7^+^ T_N_, CD4^+^CD45RA^−^ CCR7^+^ T_CM_, CD4^+^CD45RA^−^CCR7^−^ T_EM_, and CD4^+^CD45RA^+^CCR7^−^ T_TE_ cells in PBMCs from 4 HIV patients and 4 HS. **(C)** The percentages of each CD4 T cell subset within the total CD4^+^ lymphocyte populations in 4 HIV patients vs. 4 HS are shown. **(D)** PBMCs isolated from 31 HIV-infected patients and 17 HS were analyzed for T cell apoptosis by flow cytometry. Representative pseudocolor plots and summary data for the Av staining in total CD4^+^, CD45RA^+^CD4^+^ (naïve), and CD45RA^−^CD4^+^ (memory) T cells are shown. Each symbol represents an individual subject. The mean ± SEM and *p*-value are shown. Outliers in HIV groups were removed during statistical analysis. **(E)** Pearson's correlation analysis between total or memory CD4 T cell frequencies and early apoptotic cells in PBMCs isolated from HIV subjects. **(F)** Pearson's correlation analysis between CD4 T cell frequencies and actual CD4 T cell counts in HIV subjects.

Dysregulated T cell homeostasis is a characteristic of chronic viral infections (23); however, the precise mechanisms that control T cell homeostasis and virus persistence in humans remain unclear. As an initial approach to identify factors that perturb T cell homeostasis in latent HIV infection, we analyzed total (CD4^+^), naïve (CD4^+^CD45RA^+^), and memory (CD4^+^CD45RA^−^) T helper cell frequencies within peripheral blood mononuclear cells (PBMCs) isolated from HIV-infected individuals on cART with undetectable viremia or age-matched healthy subjects (HS). As shown in Figure 1A (representative pseudocolor plots and summary data of flow cytometry), the numbers of total CD4 T cells, naïve CD4, especially memory CD4 T cell pools, were significantly contracted in latently HIV-infected individuals compared to HS. To determine which subsets of CD4 T cells were most affected in latent HIV infection, we analyzed samples for their naïve CD4 (T_N_, CD4^+^CD45RA^+^CCR7^+^), central memory CD4 (T_CM_, CD4^+^CD45RA^−^CCR7^+^), effector memory CD4 (T_EM_, CD4^+^CD45RA^−^CCR7^−^), and terminal effector CD4 T cells (T_TE_, CD4^+^CD45RA^+^CCR7^−^) in this cohort. We found that T_CM_ was the primarily contracted subset in PBMCs during HIV latency (Figure 1B). We also analyzed the frequencies of these subsets in the total CD4 T cell populations from the same subjects, and observed contraction in T_CM_, but expansion in the other subsets of CD4 T cells during latent HIV infection (Figure 1C). This contraction of total CD4 T cells, particularly the T_CM_ subset, is consistent with published reports (24, 25) and indicates an alteration of T cell repertoire following cell activation, exhaustion, senescence, and apoptosis in cART-controlled, latent HIV infection.

The T cell repertoire in peripheral blood is well-maintained by a fine balance between an influx of newly generated T cells from the thymus, efflux by consumption via programmed cell death, and self-replication within the existing pool of lymphocytes. With deficient influx from the thymus in adults, the immune system responds to challenges by expanding existing T cells, leading to increased proliferative turnover, telomere erosion, and ultimately, cell apoptosis, which represents a major mechanism for controlling peripheral T cell homeostasis in adults (26). To explore how apoptosis contributes to T cell homeostasis in HIV latency, we compared the Annexin V (Av) and 7-Aminoactinomycin D (7AAD) staining in PBMCs derived from latently HIV-infected individuals and age-matched HS. As shown in Figure 1D (representative pseudocolor plots and summary data of flow cytometry), Av staining of CD4 T cells showed increases in apoptotic cells in latently HIV-infected individuals within the total CD4, as well as in naïve CD4 and, particularly, memory CD4 T cells. Importantly, apoptosis of HIV CD4 T cells, especially CD4^+^CD45RA^−^ memory CD4 T cells, appeared to be negatively correlated with their cell frequencies (Figure 1E), as analyzed by Pearson correlation. Also, the flow cytometry analysis of CD4 T cell frequencies faithfully correlated with the actual count of CD4 T cell numbers (Figure 1F), suggesting that CD4 T cell apoptosis closely correlates with CD4 T cell loss during latent HIV infection. These results indicate that the apoptotic susceptibility of CD4 T cells during latent HIV infection may necessitate compensatory homeostatic proliferation, leading to CD4 T cell exhaustion and senescence.

### CD4 T Cell Exhaustion and Senescence in cART-Controlled, Virus-Suppressed HIV Infection

**Figure 2:**
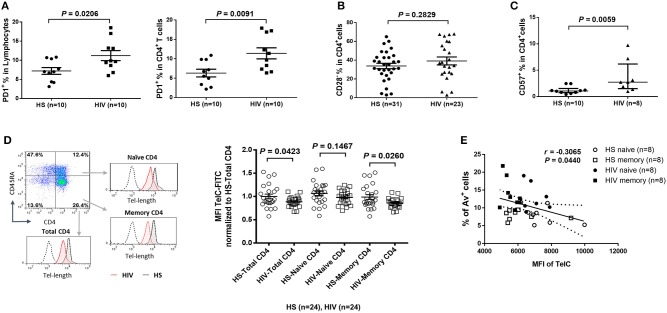
CD4 T cell exhaustion and senescence and telomere attrition in latent HIV infection. **(A)** Percentage of PD-1 expression on total lymphocytes and CD4 T cells in PBMCs isolated from 10 HIV patients vs. 10 HS, as determined by flow cytometry. **(B)** Percentage of CD28^−^ population in CD4 T cells from 23 HIV patients vs. 31 HS. **(C)** Percentage of CD57 expression on CD4 T cells from 8 HIV patients vs. 10 HS. **(D)** Representative dot plots, histogram, and summary data from 24 HIV patients and 24 HS from Flow-FISH analysis of telomere length in total, naïve, and memory CD4 T cells. **(E)** Spearman's correlation analysis of naïve and memory CD4 T cell telomere length (MFI) and the percentage of Av^+^ apoptotic cells in 8 HIV patients and 8 HS are shown.

To determine the role of homeostatic proliferation in T cell exhaustion and senescence, we first compared relevant markers expressed on CD4 T cells isolated from latently HIV-infected individuals vs. age-matched HS. As shown in Figure 2A, the expression of programmed death-1 (PD-1), a marker for cell exhaustion, was significantly upregulated in lymphocytes, as well as in CD4 T cells, in cART-controlled HIV patients compared to age-matched HS.

Since the loss of CD28, a T cell receptor (TCR) costimulatory molecule required for T cell activation and survival, is considered as an unequivocal marker of T cell senescence (27), we determined the CD28^−^ population within the CD4^+^ T cells. Similar to a report by the AGEhIV Study Group (9), we also observed a slight increase of CD28^−^ subset in CD4^+^ T cells in latently HIV-infected individuals, although it did not reach significance when compared to age-matched HS in our cohort (Figure 2B).

We have previously reported that CD57, also known as human natural killer 1 (HNK1) that is expressed on the senescent NK or T lymphocytes (28), is highly expressed on CD4 T cells derived from chronically HCV-infected individuals (20). We found that the frequency of CD57^+^ in CD4^+^ T cells was significantly increased in HIV patients vs. HS (Figure 2C). This significant upregulation of CD57 expression was also observed in CD4 T cells isolated from HIV and HS that were cultured *in vitro* for 3 days with or without TCR stimulation (*n* = 12 per group; *p* = 0.0003 and *p* = 0.0002, respectively), suggesting that HIV-derived CD4 T cells are exhausted and senescent.

### CD4 T Cell Telomere Attrition in Virus-Suppressed, Latent HIV Infection

Telomeres are repeating hexameric sequences of DNA found at chromosome ends in association with a complex of shelterin proteins. Telomere integrity is a key feature of linear chromosomes that preserves genome stability and function, whereas telomere attrition is a hallmark of cell aging or senescence that drives cell dysfunction or apoptosis (17, 18). Given the importance of telomere attrition in cell senescence, we further investigated aspects of T cell aging in HIV latency by measuring telomere length in total CD4^+^, CD4^+^CD45RA^+^ naïve, and CD4^+^CD45RA^−^ memory CD4 T cells by Flow-FISH. As shown in Figure 2D (representative plots for gating strategy and pooled data of flow cytometry), telomere length was significantly shortened in HIV-derived, total CD4 T cells, and particularly in memory CD4 T cells, compared to age-matched HS. Since telomere length is critical for cell survival, we hypothesized that longer telomeres in HS will secure cell survival, whereas shorter telomeres in HIV subjects may promote cell apoptosis. To test this hypothesis, we analyzed the relationship between cell apoptosis and telomere length in both HIV subjects and HS. Importantly, telomere length appeared to be inversely correlated with the cell apoptotic rate in naïve and memory CD4 T cells from HIV subjects and HS, as determined by Spearman correlation (Figure 2E), indicating that telomere erosion is associated with T cell apoptosis.

**Figure 3:**
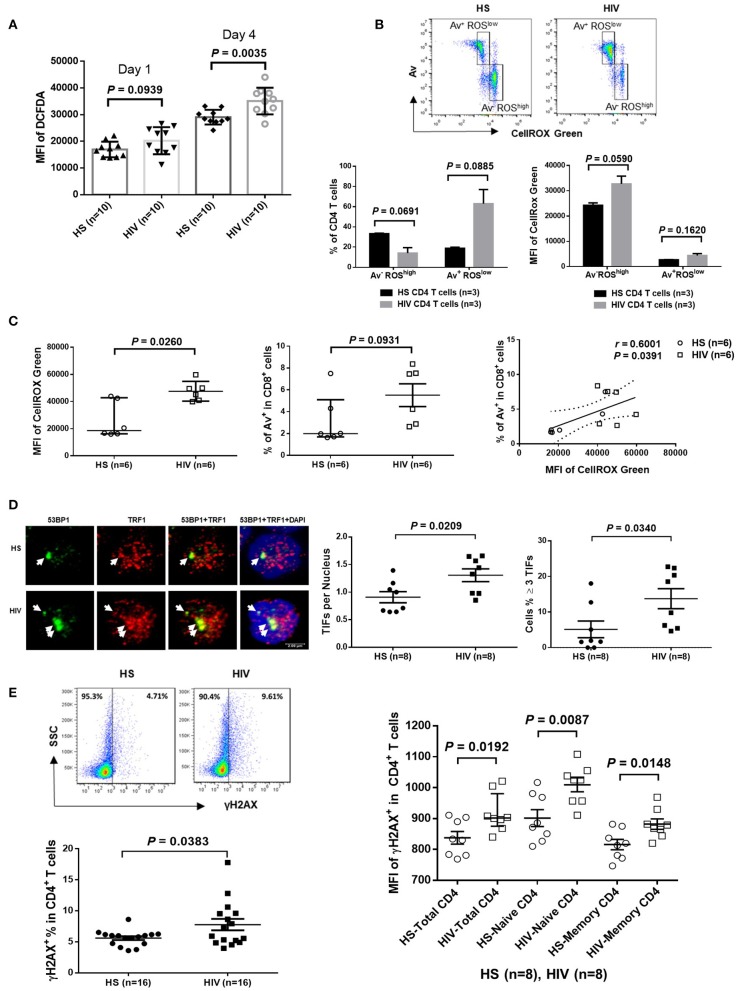
ROS generation and telomeric DNA damage in CD4 T cells from HIV patients vs. HS. **(A)** MFI of DCFDA levels in CD4 T cells isolated from PBMCs of 10 HIV patients and 10 HS, and cultured *in vitro* without stimulation for 1–4 days, then analyzed by flow cytometry. **(B)** Flow cytometry analysis for the percentage (%) and MFI of CellROX Green as well as Av levels in CD4 T cells derived from HIV or HS and cultured *ex vivo* for 4 days. Representative pseudocolor plots and summary data of the MFI as well as % of the two gated cell populations (AV^−^ ROX^high^ and Av^+^ ROX^low^) from 3 subjects in each group are shown. **(C)** MFI of CellROX Green, cell apoptotic rate, as well as the correlation of ROS production and apoptotic rate in CD8^+^ T cells in 6 HIV and 6 HS. **(D)** Confocal microscopy analysis of 53BP1 and TRF1 co-localization in the nuclei of CD4 T cells isolated from 8 HIV patients vs. 8 HS. Approximately 100 cells were counted per subject. Summary data of dysfunctional telomere-induced foci (TIF) number per nuclei and the percentage of cells with ≥3 TIFs in the two groups are shown. **(E)** Representative plots and summary data of the percentage of γH2AX expression in purified CD4 T cells from 16 HIV patients and 16 HS, as well as the MFI in total, naïve and memory CD4 T cells in PBMCs from 8 HIV patient vs. 8 HS.

Since HIV replication is well-controlled by cART in our cohort, an important question remains: what drives telomere erosion and T cell apoptosis during latent HIV infection? We and others have previously shown that naïve CD4 T cells are typically resistant to death receptor/ligand (Fas/Fas-L)-mediated apoptosis (19, 20, 29–31). Indeed, resting CD4 T cells typically do not express Fas on their cell surface, and blocking the exogenous death pathways such as Fas-Fas ligand, TNFα-TNF receptor, and TRAIL-TRAIL receptor interactions in CD4 T cells did not affect the KML001 (NaAsO2, an arsenic telomere targeting drug)-induced cell apoptosis (31), suggesting intracellular signals as initiators of apoptosis. Notably, one internal stressor linked to cell apoptosis is damaged DNA, which is particularly prominent in senescent T cells that have been chronically exposed to oxidative stress, such as endogenously generated ROS (32). To determine whether ROS might be an offender causing DNA damage and cell apoptosis during latent HIV infection, CD4 T cells were isolated from cART-controlled HIV patients and HS, and cultured *in vitro* without stimulation for 1–4 days (to generate endogenous ROS). Levels of ROS were then measured by flow cytometry using Cellular ROS Detection Kit based on the absorption of cell-permeable 2′,7′-dichloroflurescein diacetate (DCFDA)—a fluorogenic dye that measures hydroxyl, peroxyl, and other ROS activity within the cell (33). As shown in Figure 3A, the median fluorescence intensity (MFI) of DCFDA was increased in CD4 T cells derived from cART-controlled HIV patients compared to age-matched HS. Interestingly, when these cells were cultured *in vitro* without stimulation for 1–4 days, the MFI of DCFDA^high^ cells remained high in HIV T cells, whereas the percentage of DCFDA^high^ cells decreased, along with an increase in Av^+^ apoptotic cells, in HIV vs. HS (data not shown). Similar data were obtained using a different fluorogenic probe (CellROX Green) to measure ROS production in cultured CD4 T cells derived from HIV and HS. As shown in Figure 3B, depending on the levels of ROS and Av, CD4 T cells from both HIV patients and HS were gated on two major populations: Av^+^ ROS^low^ and Av^−^ ROS^high^. Notably, in both HIV patients and HS, apoptotic (Av^+^) cells produced lower amount of ROS (MFI ROS^low^) compared with non-apoptotic (Av^−^) cells (MFI ROS^high^). While the MFI of both Av^−^ ROS^high^ and Av^+^ ROS^low^ subsets remained higher in HIV than HS, the percentage of Av^−^ ROS^high^ cells was lower, whereas the percentage of Av^+^ ROS^low^ CD4 T cells was much higher in HIV patients compared to HS. Likewise, we also examined the relationship between ROS generation and cell apoptosis in CD8^+^ T cells and found that the MFI of ROS was significantly increased and positively correlated with the apoptosis levels in CD8^+^ T cells in HIV patients vs. HS (Figure 3C). These data suggest that the intracellular ROS generated during HIV infection may play an important role in triggering cell apoptosis and that the apoptotic cells produce less ROS. Av staining reflects an inversion of phosphatidylserine at the cell membrane through the activation of scramblase(s); this can be caused by other stimuli, such as type I IFN, and as such our findings are also compatible with an alternative apoptosis-independent interpretation, e.g., IFN-induced scramblase activation and anti-oxidative effects on CD4 T cells during HIV latency.

### CD4 T Cell DNA Damage in cART-Controlled, Virus-Suppressed HIV Infection

Cells are equipped with DNA damage surveillance and repair machineries to prevent cell death associated with genomic instability. Mammalian telomeres consist of triple guanine repeats (TTAGGG) that are very sensitive to oxidative DNA damage, and particularly vulnerable to ROS. We hypothesized that telomere sequences in HIV-derived T cells are not only shortened, but also DNA damaged. Notably, following genotoxic insult, p53-binding protein 1 (53BP1) is recruited to the DNA damage site on chromosomes (including telomeres) and acts as a docking site for other mediators, transducers, and DNA repair proteins to form microscopically visible nuclear foci (DNA damage foci) (34). Thus, identifying damaged telomere-induced foci (TIF) is considered as a hallmark of telomeric DNA damage response (DDR) (35). To determine telomeric DDR in T cells during latent HIV infection, we compared the number of TIFs per nucleus, as well as the percentage of cells with ≥3 TIFs, by examining co-localization of 53BP1 and telomeric repeat-binding factor 1 (TRF1) using confocal microscopy. As shown in Figure 3D (representative images and summary data), the number of TIFs per nucleus and the percentage of T cells with ≥3 TIFs were significantly higher in CD4 T cells derived from HIV patients compared to HS. Moreover, the percentage and MFI of phosphorylated H2AX (γH2AX), a marker of DNA damage, were significantly upregulated in total CD4 as well as in naïve and memory CD4 T cells from HIV patients compared to HS (Figure 3E). These results suggest that telomeric DNA damage may cause cell apoptosis and T cell loss in patients with virus-suppressed, latent HIV infection, emphasizing the role of telomere integrity in securing T cell survival.

### ATM Expression and Activity Are Inhibited in CD4 T Cells in Virus-Suppressed, Latent HIV Infection

**Figure 4:**
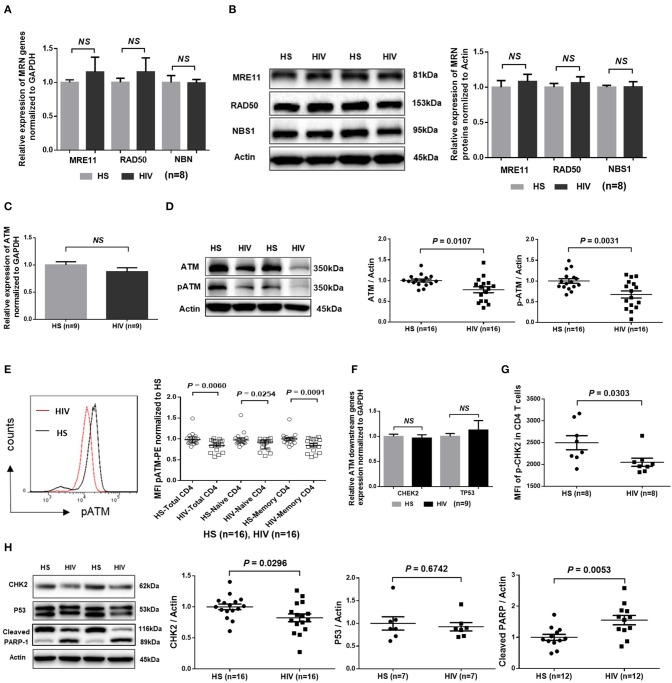
DNA damage repair ATM signaling pathway in T cells of HIV patients vs. HS. **(A)** MRE11, RAD50, and NBN mRNA expression, as determined by real-time RT-PCR, in CD4 T cells isolated from 8 latently HIV-infected patients and 8 age-matched HS. **(B)** Western blot showing MRN protein expression in CD4 T cells derived from 8 HIV patients and 8 HS. Representative images and summary data are shown. **(C)** ATM mRNA levels, determined by RT-PCR, in CD4 T cells isolated from 8 HIV patients and 8 HS. **(D)** Western blot analysis of ATM protein expression and phosphorylation in CD4 T cells derived from 16 HIV patients and 16 HS. Representative images and summary data are shown. **(E)** Flow cytometry analysis of pATM expression in total, naïve and memory CD4 T cells from 16 HIV patients and 16 HS. **(F)** CHEK2 and TP53 mRNA levels, measured by RT-PCR, in CD4 T cells from 9 HIV patients and 9 HS. **(G)** Flow cytometry analysis of pCHK2 expression in CD4 T cells from 8 HIV patients and 8 HS. **(H)** Western blot analysis of CHK2 (*n* = 16), p53 (*n* = 7), and cleaved PARP-1 (*n* = 12) levels in CD4 T cells derived from HIV patients and HS. Representative images and summary data are shown.

Accumulation of damaged DNA in CD4 T cells indicates that the DNA damage sensing and repairing machineries are impaired during HIV infection. Essential components of this machinery include DNA damage sensors, such as the MRE11, RAD50, and NBS1 (MRN) complex, which recruits and mediates activation of the DNA damage checkpoint kinase ATM that can phosphorylate several downstream checkpoint proteins, including checkpoint kinase 2 (CHK2) and p53 (36–38). To investigate the cellular machineries that promote DNA damage during HIV infection, we examined mRNA transcripts and protein expression of these DDR proteins in CD4 T cells. As shown in Figure 4A, RT-qPCR analysis of the MRN complex transcripts showed no alterations in the level of MRE11, RAD50, or NBS1. In parallel, the protein levels of these DNA damage sensors in CD4 T cells remained unchanged in HIV patients and HS (Figure 4B). Intriguingly, while the mRNA level of ATM was unchanged (Figure 4C), ATM protein level and, phosphorylation of Ser1981, an indicator of ATM activity, were inhibited in CD4 T cells from HIV patients compared to HS (Figure 4D; representative images and summary data of western blotting). The inhibition of ATM phosphorylation was confirmed by flow cytometry in total CD4 as well as in naïve and memory CD4 T cells from the studied subjects (Figure 4E). Concurrently, while the mRNA levels of the ATM downstream signaling molecule CHK2 and p53 remained unchanged (Figure 4F), phosphorylation of CHK2 (pCHK2), measured by flow cytometry, was significantly inhibited in HIV T cells (Figure 4G). CHK2 inhibition was also detected by western blot in HIV T cells compared to HS (Figure 4H). Moreover, in line with the increased DNA damage and cell apoptosis in HIV T cells, the level of full-length Poly ADP-Ribose Polymerase 1 (PARP-1) (39) was decreased, whereas the caspase 3-dependent cleaved PARP-1 was increased in HIV T cells. Taken together, these results indicate that the double-strand break (DSB) MRN sensing machinery remains intact, but the protein expression and activation of ATM/CHK2—two key components critically important for repairing DSB during DDR—are significantly suppressed at the post-transcriptional level during latent HIV infection.

### ATM Protects T Cells From DNA Damage, Cell Apoptosis, and Dysfunction via the PI3K Pathway

**Figure 5:**
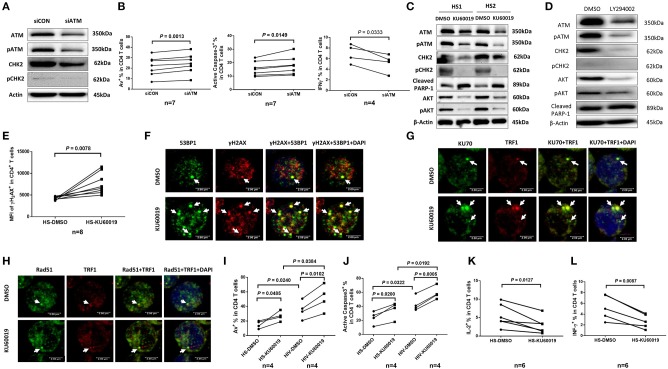
ATM plays a key role in protecting T cells from DNA damage, cell apoptosis, and cellular dysfunction. **(A)** Western blot analysis of ATM, pATM, CHK2, and pCHK2 expressions in CD4 T cells transfected with ATM siRNA (siATM) or control siRNA (siCON). Representative images from repeated experiments are shown. **(B)** Apoptosis and functional assays of CD4 T cells after ATM knockdown. Representative dot plots and summary of the percentage of Av (*n* = 7), caspase-3 (*n* = 7), and IFN-γ (*n* = 4) expressions in CD4 T cells transfected with siATM or siCON. **(C)** Western blot analysis of ATM, pATM, CHK2, pCHK2, cleaved PARP-1, AKT, and pAKT expressions in CD4 T cells treated with the ATM inhibitor (KU60019) or DMSO for 48 h. **(D)** Western blot analysis of ATM, pATM, CHK2, pCHK2, AKT, pAKT, and cleaved PARP-1 expressions in CD4 T cells treated with the PI3K inhibitor (LY294002) or DMSO for 48 h. Representative images from repeated experiments are shown. **(E)** Flow cytometry analysis for the MFI of γH2AX expression in CD4 T cells treated with KU60019 or DMSO for 48 h (*n* = 8). **(F–H)** Representative images of confocal microscopy examination of co-localization of 53BP1 and γH2AX, Ku70 and TRF1, RAD51 and TRF1, in CD4 T cells treated with KU60019 or DMSO for 48 h (*n* = 6). **(I,J)** Flow cytometry analysis of Av% and caspase-3% in CD4 T cells from HIV and HS exposed to KU60019 or DMSO for 48 h (*n* = 4). **(K,L)** Summary data of IL-2 and IFN-γ productions in healthy CD4 T cells stimulated with PMA plus ionomycin and blocked with brefeldin A for the last 5 h in the presence of KU60019 or DMSO for 48 h (*n* = 6).

The ATM signaling pathway is pivotal to the maintenance of genome integrity and cell survival (40, 41). To determine the role of ATM in T cell function and survival, we performed siRNA-mediated knockdown in healthy CD4 T cells, and then measured cell apoptosis and cytokine expression. As shown in Figure 5A, healthy CD4 T cells transfected with siRNA specific to ATM (siATM) exhibited a significant decrease in ATM and pATM protein levels compared to those treated with control siRNAs (siCON). Concurrently, CHK2 and pCHK2 expressions were also diminished in ATM-silenced T cells. In addition, the frequencies of Av^+^ and active caspase-3^+^ apoptotic cells were markedly increased, whereas the number of IFN-γ-producing CD4 T cells was significantly decreased (Figure 5B) after ATM knockdown.

To further investigate the role of ATM in maintaining T cell survival and function, primary CD4 T cells isolated from HS were treated with a specific ATM inhibitor (ATMi, KU60019) for 48 h, followed by measuring expression and activation of the ATM/CHK2 pathway and cell apoptosis. As shown in Figure 5C, healthy CD4 T cells exposed to KU60019 had a marked decrease in the expression of ATM, and in particular, pATM, compared to the DMSO-treated control. Concurrently, the levels of ATM downstream signaling molecule CHK2, and particularly pCHK2, were significantly suppressed by this treatment. In addition, T cells exposed to the ATMi exhibited an elevated level of cleaved PARP-1, indicating increased caspase 3-dependent, DNA damage-mediated cell apoptosis.

ATM belongs to the phosphoinositide 3-kinase (PI3K) family and plays a key role in cell survival and function (40, 41). We thus examined whether the expression and activation of AKT (also known as protein kinase B) could be suppressed by the ATM inhibition. As shown in Figure 5C, both AKT and pAKT were inhibited in CD4 T cells exposed to the KU60019 treatment. In parallel, we also examined whether ATM expression and activation could be blocked by the PI3K inhibitor (PI3Ki). To this end, we incubated CD4 T cells with or without the PI3Ki (LY294002) for 48 h and measured ATM/pATM, CHK2/pCHK2, AKT/pAKT, as well as cleaved PARP-1 expressions by western blot. As shown in Figure 5D, compared to the DMSO control, T cells treated with 20 μM of LY294002 exhibited significantly lower ATM/pATM and CHK2/pCHK2 expressions, along with suppression of AKT/pAKT, and an increase in the level of cleaved PARP-1. These results indicate cross-talk between the ATM and AKT pathways, and that ATM deficiency is likely induced via inhibition of the PI3K pathway.

### The Non-homologous End Joining (NHEJ) Pathway Is Involved in ATM-Mediated DNA Repair and Telomere Maintenance

We next examined the role of ATM in protecting T cells from DNA damage and found that T cells treated with the ATMi exhibited an increase in the expression of γH2AX, a marker for DNA damage (Figure 5E). These T cells also had increases in DNA damage foci (γH2AX/53BP1 co-localization), as measured by confocal microscopy (Figure 5F). It is well-known that the Ku70 and Ku80 heterodimer binds to DSB ends and is required for NHEJ-mediated DNA repair. It is also required for V(D)J recombination, which utilizes the NHEJ pathway to promote antigen diversity in the mammalian immune system. In addition to its role in NHEJ, Ku is also involved in telomere maintenance (42). It has been reported that mutant mice with deficient Ku70 exhibit premature aging (43), suggesting that Ku70 plays an important role in longevity assurance and that reduced ability to repair DSB or maintain telomere integrity causes premature aging. To determine the role of ATM in Ku70-mediated telomeric DNA damage repair, we measured the Ku70-dependent, damaged telomere-induced foci (TIF) in CD4 T cells exposed to ATMi for 48 h using confocal microscopy. As shown in Figure 5G, Ku70/TRF1-formed TIFs were markedly increased in cells treated with the ATMi compared to the DMSO-treated control. We also examined the possible involvement of the ATM pathway in homologous recombination (HR) of DNA during DSB repair. Since RAD51 is involved in the search for homology and strand pairing process (44, 45), we used confocal microscopy analysis for the RAD51/TRF1-dependent TIF in CD4 T cells exposed to ATMi or DMSO for 48 h, but found no significant difference in TIFs between the two treatments (Figure 5H). These results suggest that the NHEJ pathway, rather than the HR pathway is involved in ATM-mediated telomeric DNA repair.

Next, we examined the role of ATM in protecting T cells from cell apoptosis and cellular dysfunction. We found that ATM inhibition in CD4 T cells increased T cell apoptotic death and that HIV T cells appeared to be more vulnerable to the ATMi-mediated apoptosis, as evidenced by the significantly higher baseline and the level of increases in Av staining (Figure 5I) as well as active caspase-3 levels (Figure 5J) in CD4 T cells derived from HIV patients vs. HS. Meanwhile, these ATMi-treated CD4 T cells exhibited diminished IL-2 (Figure 5K) and IFN-γ expressions (Figure 5L) compared to the controls. Taken together, these results demonstrate that ATM inhibition can lead to increased DNA damage, cell apoptosis, and cell dysfunction, all of which potentially contribute to the CD4 T cell loss and poor immune responses that are observed in latent HIV infection.

### Ectopic Expression of ATM in HIV CD4 T Cells Reduces DNA Damage, Cell Death, and Cellular Dysfunctions

**Figure 6:**
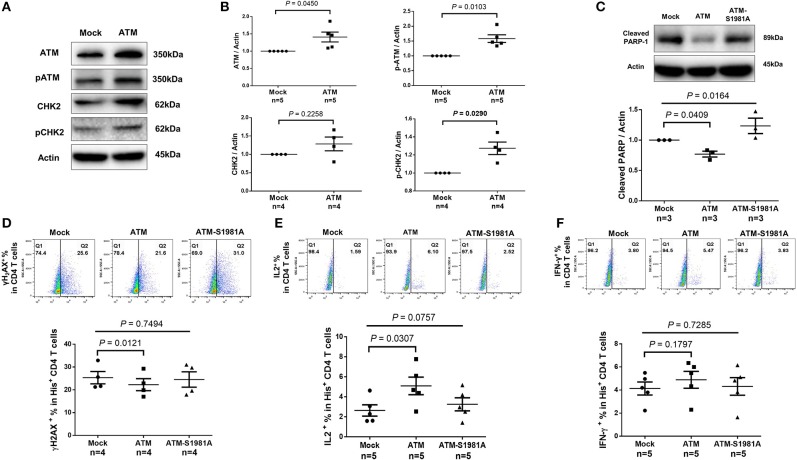
Ectopic expression of ATM reduces DNA damage, cell apoptosis and dysfunction of CD4 T cells from HIV patients. **(A–C)** Western blot analysis of ATM/pATM (*n* = 5), CHK2/pCHK2 (*n* = 4), and cleaved PARP-1 (*n* = 3) expression in CD4 T cells derived from HIV-infected patients with or without ATM overexpression. **(D–F)** Representative dot plots and summary data of flow cytometry analysis of γH2AX (*n* = 4), IL-2 (*n* = 5), and IFN-γ (*n* = 5) expressions in Mock-, ATM-, and ATM-S1981A-transfected (His^+^) CD4 T cells derived from HIV patients. *P*-values were determined by paired *t*-tests for two groups and by one-way ANOVA for three groups.

Given the critical role of ATM in repairing DNA damage, we hypothesized that the ectopic ATM could recover HIV T cells from DNA damage and restore the signaling network required for repairing DNA-DSB. To test this, we transfected CD4 T cells derived from HIV-infected, cART-controlled individuals with Flag-His-ATM expression construct or control plasmids; an empty vector without ATM insert (Mock), and an ATM kinase-dead mutant (ATM-S1981A) in which the serine residue at 1981 was substituted by an alanine residue. As shown in Figure 6A, western blot analysis confirmed the increases in ATM and pATM expressions in the ATM-transfected T cells compared to the control. Additionally, protein expression and phosphorylation (pCHK2) of the ATM downstream signaling molecule CHK2 were increased in ATM-transfected cells (Figure 6B). Although ectopic overexpression of ATM could have broad biological effects, we focused our investigation on DNA repair, cell survival, and cell function by assessing cleaved PARP-1, caspase-3, γH2AX, IL-2, and IFN-γ expressions as readouts. Specifically, the caspase-3-dependent cleavage of PARP-1 was decreased in T cells transfected with the wild-type ATM, but not with the ATM kinase-dead mutant (Figure 6C). Concurrently, the level of the active form of caspase-3 was reduced in T cells with ATM reconstitution, but not with the ATM-S1981A transfection, though such decrease was not significant (data not shown). Compared to the mock transfection, overexpression of ATM significantly reduced γH2AX level in transfected (His^+^) CD4 T cells, whereas transfection of the ATM-S1981A mutant did not elicit such an effect, indicating that the ATM kinase activity is important for protecting cells from excessive DNA damage (Figure 6D). These results are in line with the data showing that expression of wild-type ATM significantly reduced cleaved PARP-1, whereas mock- or ATM-S1981A-transfected cells exhibited relatively higher levels of cleaved PARP-1 (Figure 6C). These results suggest that ectopic ATM expression exerts immediate effects on repairing DNA damage and regulating T cell fate to secure cell survival. Importantly, the reconstitution of ATM restored T cell function, as demonstrated by the significant increase in IL-2 (Figure 6E) and the partial recovery of IFN-γ expression (Figure 6F) in ATM-transfected cells, whereas ATM-S1981A transfection could not induce such an effect. Taken together, these results demonstrate that restoring an adequate wild-type ATM level in CD4 T cells during cART-controlled, latent HIV infection is necessary and sufficient to reduce the DNA damage, cell death, and cell dysfunctions.

## Discussion

**Figure 7:**
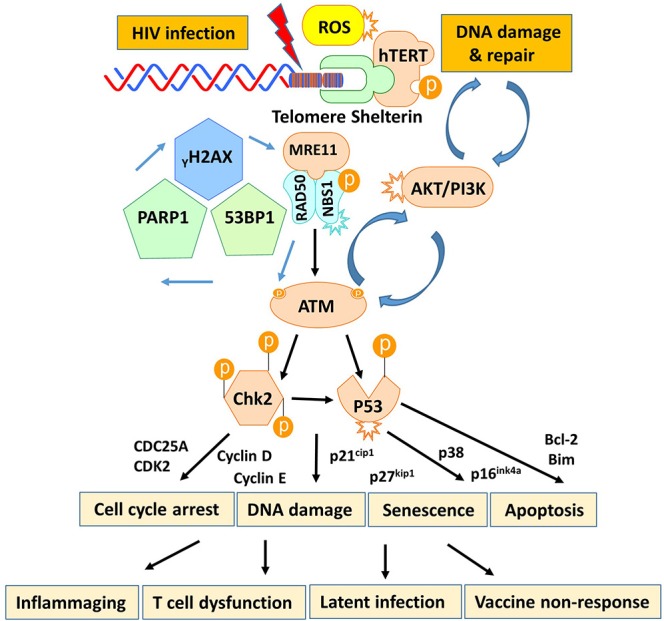
A working model depicting the role of HIV-induced ATM deficiency in T cell cycle arrest, telomeric DNA damage, cell senescence, and apoptosis during chronic viral infection. HIV infection triggers the DNA damage response (DDR) during the early phase of infection via activation of the MRN-ATM-CHK2/P53 signaling pathways in CD4 T cells, promoting cell cycle arrest (through the p21/p27-regulated CDK/Cyclins) and allowing DNA damage repair. If the infection persists and causes irreparable DNA damage, the cell will commit suicide and undergo apoptosis. Only a very small population of HIV reservoirs harbor provirus. However, the persistent, low grade of antigenic and inflammatory or ROS stimulation in the setting of latent HIV-1 infection in individuals on cART drives the majority of uninfected, bystander T cell exhaustion (PD-1) and senescence (telomere attrition) by inducing ATM (PI3K-like kinase) activation/exhaustion and deficiency (low protein expression and phosphorylation). This leads to inflammaging and diminished DNA damage repair, which results in aberrant DNA damage (γH2AX, 53BP1 accumulation), cell apoptosis (caspase-3-dependent cleavage of PARP-1), and thus, constant CD4 T cell depletion. Excessive CD4 T cell loss necessitates homeostatic proliferation and imposes replicative stress on reactive, memory CD4 T cells as well as unprimed, naïve CD4 T cells, leading to accelerated, premature T cell aging or senescence. This working model represents a novel molecular mechanism underlying the T cell dysfunction and immune (vaccine) non-responsiveness seen in the setting of latent HIV infection.

T cells play a pivotal role in controlling viral infection. However, the mechanisms that dysregulate T cell functions during viral infection, particularly in the latent phase, remain incompletely understood. In this study, we investigated the mechanisms that perturb T cell homeostasis, apoptosis, DNA damage and repair machineries in CD4 T cells from patients with cART-controlled, latent HIV infection. We found a significant depletion of CD4 T cells, which was inversely correlated with cell apoptosis in HIV-infected individuals on cART. Additionally, HIV-derived CD4 T cells exhibited an accumulation of DNA damage that extended to telomeres, leading to accelerated telomere attrition. Mechanistically, the DNA damage sensor MRN complex remained intact, but the expression and activation of PI3K family member ATM and its downstream checkpoint kinase CHK2 were significantly suppressed in HIV T cells. Consistently, ATM/CHK2 activation and cellular functions were impaired in primary healthy CD4 T cells following ATM silencing or exposure to an ATM inhibitor *in vitro*, which recapitulated the biological effects we observed *in vivo* in HIV-infected patients on cART. Importantly, ectopic expression of ATM was essential and sufficient to reduce the DNA damage, survival deficit, and cell dysfunctions in HIV-derived T cells. These results demonstrate that ATM deficiency can lead to accumulation of telomeric DNA damage, rendering CD4 T cells prone to apoptosis in latent HIV infection. Based on these findings, we propose a novel model (depicted in Figure 7) in which HIV infection and/or chronic inflammation-induced ATM deficiency drives accumulation of telomeric DNA damage, premature T cell senescence, and apoptosis. This aberrant and constant CD4 T cell depletion would necessitate high homeostatic proliferation that imposes replicative stress on naïve and particularly memory CD4 T cells, leading to further cell senescence or apoptosis—a malicious cycle for CD4 T cell dysregulation in latent HIV infection.

The mechanisms underlying inflammaging in cART-controlled, latent HIV infection remain elusive, but multiple factors may be involved. Specifically, HIV latency in the era of cART is characterized by the existence of viral reservoirs that prevent HIV-1 eradication and likely drive inflammaging (46, 47). Thus, HIV-mediated inflammaging could result from a myriad of insults, such as viral particles or viral RNAs/proteins released from the reservoirs, cell-secreted pro-inflammatory cytokines, HIV-enhanced gut permeability and altered gut microbiota or dysbiosis, frequent cytomegalovirus (CMV), Epstein–Barr virus (EBV), hepatitis B virus (HBV), and HCV coinfections, the cART regimen itself (NRTIs may take part in telomere damage and T cell senescence, as telomerase has been shown to be inhibited by some NRTIs) (48), as well as other comorbidities including malignancies, personal stresses, or social and environmental factors (49–52). In addition to central memory CD4 T cells, several groups have reported that T stem cell memory (T_SCM_) cells harbor HIV-1 provirus and are relatively “spared” from depletion as compared to other CD4 T cells (53, 54). Since T_SCM_ cells fall under the naïve population (CD45RA^+^), and this minor subpopulation is characterized by its resistance to apoptosis, increased proliferation, and lower ROS levels, skewing T_SCM_ cell activation and/or differentiation into T_CM_/T_EM_/T_TE_ CD4 T cells might partly help to explain why T cell senescence is increased in the absence of active HIV replication. Since CD4 T cells from cART-controlled, latent HIV infection are mostly uninfected and have an extremely low number of cells harboring provirus (only 1–2 cells have HIV-1 per million of CD4^+^ T cells) (55), the observed results suggest overall cellular senescence is more likely related to cell exhaustion associated with increased immune activation and over-proliferation driven by chronic inflammation during HIV latency. As such, the cellular clock that determines the number of cell divisions is constantly turned “on,” causing critical telomere shortening and eventually inhibiting further cell divisions. As demonstrated in this study, ROS generated within the cellular milieu during viral infection might also drive inflammaging. Thus, the observed effects are likely related to chronic inflammation, rather than the HIV reservoir itself, and such cellular exhaustion or senescence could also apply to other chronic infections, inflammatory diseases, and cancers. Interestingly, this residual chronic, low-grade inflammation-mediated inflammaging during latent HIV infection appears to trigger immune activation that may increase the risk of developing comorbidities, similar to those observed in the elderly. For example, individuals living with HIV have accentuated risks for age-associated comorbidities: a two-fold higher risk of cardiovascular disease, a three-fold increased risk of fracture, and a risk of developing kidney disease that is comparable to those with diabetes (8). Some comorbidities may present at younger ages compared to the general population, suggesting accelerated, premature aging in the setting of well-controlled but latent HIV infection (56).

An important question is why and how CD4 T cells accumulate DNA damage and fail to repair telomere defects during HIV infection? Human CD4 T cells have a relatively long-life span (~150–160 days) and thus are exposed to a multitude of genotoxic stresses, leading to ~1% out of a pool of 300 billion T cells being replaced daily (26). To maintain genomic stability and cell survival, cells continuously recognize and respond to DNA damage either via activation of DNA damage checkpoints to arrest cell cycle progression and allow for repair or, if the damaged DNA is beyond repair, by promoting apoptosis. Notably, a major sensor of DSB is the MRN complex, which subsequently recruits the protein kinase ATM, an enzyme critically involved in repairing DNA damage for cell survival (40, 41). Our results revealed that while the MRN complex was intact, ATM was dramatically affected by HIV, even in the setting of a robust viral suppression, and was associated with significant DNA damage at telomeres. Accumulation of damaged DNA activates the ATM cascades, along with ataxia-telangiectasia Rad3-related (ATR) and DNA-dependent protein kinase catalytic subunit c (DNA-PKc), which all belong to the PI3K-related kinase (PIKK) family and are important for DNA reprogramming and T cell rearrangement (40, 41). Because the immune system is in constant turnover during viral infection, with high demands for lymphocyte replenishment to maintain the equilibrium of the T cell compartment, insufficient activation of ATM would be expected to affect both primed and unprimed T cells. However, memory T cells turnover rapidly with greater DNA damage and cell apoptosis, which could compromise the size and survival rate of the naïve T cell repertoire. Indeed, ongoing antigenic and/or inflammatory stimulation during chronic viral infection induces continuous differentiation of the naïve T cells and turnover of antigen-reactive T cells, resulting in telomere erosion and cell senescence or apoptosis, which we observed more prominently in memory T cells. Eventually, the majority of the T cell pool would be comprised of antigen-expanded T cells at the expense of naïve T cells. With the decrease in newly generated naïve thymic T cells in adults, chronic inflammation or infection might force the immune system to restore equilibrium by replicating the existing naïve T cells, thereby driving telomere shortening and senescence in naïve T cell populations in the peripheral blood. Thus, the ability to generate an immune response to new antigens, such as vaccines, would be compromised.

ATM was originally identified in individuals with ataxia-telangiectasis (AT), an autosomal recessive disorder that manifests with progressive ataxia, telangiectasia, immunodeficiency, genomic instability, and cancer predisposition (57). It is a key kinase within the DDR signaling cascade and plays a unique role in lymphocyte biology, as programmed DDR participates in the gene remodeling process necessary for the formation of a highly diverse TCR repertoire (39, 40). ATM requires an activation signal (usually a DNA damage signal), which promotes intermolecular auto-phosphorylation at ATM residue at serine 1981, resulting in the dissociation of the inactive dimer into active monomers (58). In the current study, a mutant of S1981A rendered ATM a dominant-negative protein and triggered distinct DNA damage-mediated cell apoptosis and cellular dysfunction (e.g., IL-2 inhibition), which underscored the importance of ATM phosphorylation for its biological functions. ATM is predominantly localized in the nucleus, and is activated once the MRN complex senses and binds to DSB ends, thus providing a platform for ATM recruitment and auto-phosphorylation (59, 60). Phosphorylation of S1981 also stabilizes ATM at the damaged DNA sites and recruits more downstream effector proteins to participate in the DDR (61). Among the multiple substrates phosphorylated by ATM is CHK2, which is phosphorylated at residue T62 following DSB formation and prevents the cell cycle from progressing from the G1 to the S phase or, alternatively, leads to cell apoptosis. Our results showed that concomitant with the ATM deficiency, CHK2 phosphorylation was defective in CD4 T cells during HIV infection. Moreover, siRNA-mediated silencing or pharmacological inhibition of ATM in healthy T cells also led to the CHK2 phosphorylation defect, which was accompanied by dramatic DNA damage and cell apoptosis—mimicking the biological defect we observed in HIV-derived CD4 T cells. These results establish that in human T cells, CHK2 is a downstream target of ATM and that the overall defect of this signaling pathway can be attributed to ATM insufficiency, driven by chronic inflammation, or oxidative stress in latent HIV infection. Further investigations of the molecular mechanisms regulating the post-transcriptional expression, phosphorylation, and/or dephosphorylation of these DDR kinases will shed new light on the cellular machinery that contributes to DNA repair and cell homeostasis.

Notably, the DNA damage sensor MRN complex is upstream of ATM activation. It has been shown that patients with rheumatoid arthritis exhibit premature T cell aging with damaged telomeres and defective activity of the DNA break sensor MRE11A (62). The DNA repair nuclease MRE11A also functions as a mitochondrial protector and prevents T cell pyroptosis and tissue inflammation (63). In patients with latent HIV infection, however, we found an intact MRN complex in CD4 T cells. Of note, ATM was inhibited at the post-transcriptional level in these cells, which produced considerable amounts of ROS. Interestingly, Guo et al. also reported that ATM activation in response to ROS was independent of the MRN complexes (64, 65). ROS-mediated ATM signaling represses mTORC1 signaling and, therefore also inhibits cell growth and proliferation through the activation of TSC2 (a negative regulator of mTOR) by liver kinase B1 (LKB1) and AMP-dependent protein kinases (AMPKs) (66). Also, ATM engagement of the TSC2/mTORC1 signaling pathway can regulate autophagy, and differential localization of ATM correlates with activation of distinct downstream signaling pathways (67, 68). Likewise, we have recently reported that T cells derived from HCV infection also exhibit pronounced telomeric DNA damage due to remarkably elevated ROS, with intact DNA damage sensors MRN complex but impaired ATM/CHK2 signaling pathway (19, 20). Investigation is underway in our laboratory to illustrate the detailed mechanisms of ROS-mediated ATM deficiency during latent HIV infection.

It should be pointed out that ATM, as a key kinase in DDR, can be activated in the early phase of DNA damage, as observed in our *in vitro* model, in which pATM, pCHK2, and pAKT were upregulated, whereas their total protein levels were downregulated in CD4 T cells following active HIV infection (unpublished observation). These changes led to a global loss of ATM, CHK2, and AKT, which suggests an activation and a subsequent exhaustion or senescence process. Notably, ATM is widely expressed in human T cells at a high level to ensure the integrity of genomic DNA in replicating lymphocytes. Its activation may represent the initiation of DDR, but its inhibition and insufficiency in persistently activated T cells indicate a deficiency in DNA repair that can promote cell exhaustion and senescence in the setting of chronic viral infection. This notion is also supported by our recent observations in the *in vitro* stimulated T cell system in which ATM phosphorylation was increased in the early phase (3–6 h) of T cell treatment with KML001 (a telomere targeting drug), or CPT (a topoisomerase I inhibitor), but decreased with longer treatment period (24–48 h), and was associated with increases in DNA damage, cell apoptosis, and functional impairment (31, 69).

As noted above, our data demonstrated that both ATM and CHK2 expressions and activations were ultimately diminished, along with telomeric DNA damage and cell apoptosis in the setting of cART-controlled, latent HIV infection. HIV becomes suppressed and inactive within infected cells or reservoirs, but still drives the inflammatory changes in the bystander cells during latent infection. These findings beg the question of whether it is even feasible to avoid disruption of telomeric DDR signaling and cellular senescence/apoptosis in the HIV-infected host without actually targeting the repair machinery itself. Consistent with our results and proposed concept, Rossiello et al. and Fumagalli et al. reported that telomeric DNA damage in mammalian cells is irreparable and occurs preferentially at telomeres in linear genomes (70, 71). Their studies suggested a paradigm in which cellular senescence and organismal aging resulting from persistent DNA damage signaling and accumulated, irreparable telomeric DNA damage could be permanent via a shared causative mechanism; i.e., once damaged, they remain damaged. Other data suggest that persistent DDRs may trigger senescence-associated inflammatory cytokine secretion, and thus may facilitate communication of a compromised state to the surrounding cells (72). Similarly, a bystander effect could occur in which senescent cells induce DDR in proximate cells within the milieu via cell-cell contact and ROS generation, a notion of senescence-induced senescence (73). These mechanisms could certainly occur in this model of HIV-infected individuals on cART. Importantly, latently HIV-infected cells are more susceptible to DDR-inducing agents, and thus are vulnerable to DDR-induced cell apoptosis (74). Sensitivity to DDR highlights a unique vulnerability of latently infected cells, a new feature that could potentially be exploited in developing therapeutics to eliminate HIV reservoirs. Further studies to explore the DDR triggers are underway in our laboratory.

In summary, the accumulation of DNA damage and failure to repair mechanisms due to deficiency of the ATM-dependent DNA repair machinery during chronic viral infection may broadly impair diverse cellular functions. Counteracting ATM deficiency may restore T cell homeostasis and competency during chronic viral infection and prevent premature T cell aging or immune senescence. This study reveals a novel molecular mechanism underlying T cell aging and provides a new strategy to develop innovative approaches to correct an aberrant immunopathology, to avoid the untoward consequences of immune senescence and to improve immunotherapy and vaccine responses against human viral diseases.

## Materials and Methods

### Subjects

The study protocol was approved by the institutional review board (IRB) of East Tennessee State University and James H. Quillen VA Medical Center (ETSU/VA IRB, Johnson City, TN). The study subjects were composed of two populations: 183 ART-controlled HIV patients and 185 age-matched HS. HIV subjects were virologically suppressed for HIV replication with cART, as evidenced by an undetectable level of HIV-RNA. Healthy subjects supplied by Physicians Plasma Alliance (Gray, TN) were negative for HBV, HCV, and HIV infection. All participants were adults and signed an informed consent form.

### Cell Isolation and Culture

Peripheral blood mononuclear cells (PBMCs) were isolated from whole blood by Ficoll density centrifugation (GE Healthcare, Piscataway, NJ). CD4^+^ T cells were isolated from PBMCs using the CD4^+^ T Cell Negative Selection Kit (Miltenyi Biotec Inc., Auburn, CA). T cells were cultured in RPMI 1640 medium containing 10% FBS (Atlanta Biologicals, Flowery Branch, GA), 100 IU/ml penicillin, and 2 mM L-glutamine (Thermo Scientific, Logan, Utah) at 37°C and 5% CO_2_ atmosphere. Cells were harvested after day 1 or day 4 of culture for the detection of apoptosis and DNA damage. To test the role of ATM activation in repairing DNA damage and apoptosis, 10 μM ATM inhibitor (KU60019, Abcam, Cambridge, MA) or DMSO were added to the cultures at day 1, and the cells were collected after 48 h for measuring apoptosis and DNA damage. To determine the role of the PI3K pathway in ATM activation, purified CD4 T cells were cultured for 48 h in the presence or absence of 20 μM PI3K inhibitor (LY294002, Sigma), followed by flow cytometry or western blot analysis.

### Flow Cytometry

For phenotypic analysis of CD4 T cells, PBMCs were stained with CD4-FITC, CD8-PE, CD57-APC (BioLegend, San Diego, CA), CD28-PE (Invitrogen, Carlsbad, CA), CD45RA-PerCP710, PD1-FITC (eBioscience, San Diego, CA), or isotype control antibodies. To quantify cell apoptosis, PBMCs were stained with CD4-A647 and CD45RA-FITC for naïve or memory cell populations, and then stained with Annexin V-PE and 7-AAD using BD Pharmingen™ PE Annexin V Apoptosis Detection Kit I (BD Biosciences, San Jose, CA). CD4^+^ T cells were also stained for caspase-3 expression following a protocol from CaspGLOW™ Fluorescein Active Caspase-3 Staining Kit (Invitrogen). Levels of reactive oxygen species (ROS) in CD4 T cells were measured using the DCFDA-based Cellular ROS Detection Kit (Abcam, Cambridge, MA) or CellROX Green ROS Detection kit (ThermoFisher Scientific, Waltham, MA) according to manufacturer's protocol. For intracellular staining, the cells were fixed and permeabilized with Foxp3 Transcription Factor Staining Buffer Set (eBioscience), and stained with pATM (Ser1981)-PE antibody (BioLegend), pCHK2 (Thr68)-PE antibody, γH_2_AX-PE (eBioscience), IL-2-PE, and IFN-γ-PE (Invitrogen). Flow cytometry was carried out as described previously (19, 20).

### Flow-FISH

Telomere length was measured by a modified Flow-FISH method as described previously (20). Briefly, PBMCs were stained with CD4-A647 and fixed with Cell Fixation buffer (BioLegend) for 20 min. The cells were incubated with a telomere probe TelC (CCCTAACCCTAACCCTAA)-FITC (0.25 μg probe/mL, PNA Bio, Newbury Park, CA) at room temperature for 10 min in dark, and then at 82°C for 10 min, room temperature overnight. The cells were washed with post-hybridization buffer twice, and then stained with CD45RA-perCP710 and analyzed by flow cytometry.

### RNA Isolation and Real-Time RT-PCR

Total RNA was extracted from 1 × 10^6^ cells with PureLink RNA Mini Kit (Invitrogen), and cDNA was synthesized using High Capacity cDNA Reverse Transcription Kit (Applied Biosystems, Foster City, CA) per the manufacturer's instruction. Quantitative RT-PCR was performed in triplicates using the following conditions: 1 cycle at 95°C for 10 min, followed by 40 cycles at 95°C for 15 s; 60°C for 60 s; and 72°C for 60 s. Gene expression was normalized to GAPDH, and values were calculated using the 2^−ΔΔ*ct*^ method and are expressed as fold changes. The PCR primers are shown in Table 1.

**Table 1:** The PCR primers used in this study.

**Genes to be amplified**	**Primer sequences**
ATM	F: 5′-TGGATCCAGCTATTTGGTTTGA-3′
	R: 5′-CCAAGTATGTAACCAACAATAGAAGAAGTAG-3′
MRE11	F: 5′-CTTGTACGACTGCGAGTGGA-3′
	R: 5′-TTCACCCATCCCTCTTTCTG-3′
RAD50	F: 5′-CTTGGATATGCGAGGACGAT-3′
	R: 5′-CCAGAAGCTGGAAGTTACGC-3′
NBN	F: 5′-TTGGTTGCATGCTCTTCTTG-3′
	R: 5′-GGCTGCTTCTTGGACTCAAC-3′
CHEK2	F: 5′-CCCAAGGCTCCTCCTCACA-3′
	R: 5′-AGTGAGAGGACTGGCTGGAGTT-3′
TP53	F: 5′-TCAACAAGATGTTTTGCCAACTG-3′
	R: 5′-ATGTGCTGTGACTGCTTGTAGATG-3′
GAPDH	F: 5′-TGCACCACCAACTGCCTTAGC-3′
	R: 5′- GGCATGGACTGTGGTCATGAG-3′

### Western Blotting

Western blot was performed as described previously (19, 20), using pATM (Ser1981) (D6H9), MRE11, RAD50, NBS1, PARP-1, pAKT pCHK2 (Thr68) (C13C1), γH2AX (Cell Signaling, Danvers, MA), and β-Actin (8H10D10) antibodies (Cell Signaling). Appropriate horseradish peroxide-conjugated secondary antibodies (Cell Signaling) were used, and proteins were detected with the Amersham ECL Prime Western Blotting Detection Reagent (GE Healthcare Bio-Sciences, Pittsburgh, PA). The blotted membranes were stripped and re-probed with ATM (D2E2), AKT, and CHK2 (D9C6) antibodies (Cell Signaling). Protein bands were captured and quantified by Chemi Doc^TM^ MP Imaging System (Bio-Rad).

### Confocal Microscopy

CD4^+^ T cells were isolated and cultured as described above, followed by immunofluorescence staining using a method described previously (19, 20). Primary antibodies included: rabbit anti-53BP1, anti-RAD51, anti-KU70 (Cell Signaling) and mouse anti-γH_2_AX (Ser139) (Biolegend), or mouse anti-TRF1 (Thermo Fisher). Secondary antibodies included: anti-rabbit IgG-Alexa Fluor 488 and anti-mouse IgG- Alexa Fluor 555 (Invitrogen). The cells were mounted with DAPI Fluoromount-G (SouthernBiotech, Birmingham, AL), and images were acquired using a confocal laser-scanning inverted microscope (Leica Confocal, Model TCS sp8; Germany).

### ATM Silencing

Approximately, 1 × 10^7^ CD4 T cells were isolated from HS and transfected with 100 pmol of ATM-specific SMARTpool siGENOME siRNAs or scrambled siRNAs (Dharmacon, Lafayette, CO) using the Human T Nucelofector Kit and Nucleofector I Device with program U-14 (Lonza, Allendale, NJ). After 48 h, the cells were harvested and analyzed by flow cytometry, western blot, and confocal microscopy.

### ATM Overexpression

Purified CD4^+^ T cells from HIV patients were transfected with 2.5 μg of pcDNA3.1, pcDNA3.1(+)Flag-His-ATM wt or human mutant ATM [S1981A; Addgene plasmid # 31985 or 32300, a gift from Michael Kastan (58, 75)], using the Human T Nucleofector Kit and Nucleofector I Device (Lonza, Allendale, NJ). After 48 h, transfection efficiency was monitored by flow cytometry to measure the frequency of His^+^ cells. Ectopic ATM expression was detected by western blot. Active caspase-3, γH_2_AX, and IL-2 expressions were assessed in the His-positive cells by flow cytometry.

### Statistical Analysis

The data were analyzed using Prism 7 software and are presented as mean ± SEM or median with interquartile range, depending on if the values pass the D'Agostino-Pearson normality or Kolmogorov-Smirnov test. Statistical outliers were removed by ROUT method (Q = 1%). Differences between two groups were analyzed by independent Student's *t*-test or Mann Whitney *U*-test, paired *T*-test or Wilcoxon matched-pairs signed-rank test. Differences among three groups were analyzed by one-way ANOVA test. Correlations were analyzed by either Pearson's or Spearman's correlation, depending on the data normal distribution test. *P* < 0.05, or <0.01 were considered statistically significant or very significant, respectively.

## Data Availability Statement

All datasets generated for this study are included in the article/supplementary material.

## Ethics Statement

The study protocol was approved by the institutional review board (IRB) of East Tennessee State University and James H. Quillen VA Medical Center (ETSU/VA IRB, Johnson City, TN). All participants were adults and signed the informed consent form.

## Author Contributions

JZ performed most of the experiments. LNTN, LNN, XD, DC, SK, MS, BT, SO, and LW participated in some experiments. XW and ZM provided technical support. ZL, YZ, ME, SN, and JM offered intellectual input for troubleshooting and discussion of the findings. ZY supervised the project and wrote the manuscript, with the help of all other authors.

### Conflict of Interest

The authors declare that the research was conducted in the absence of any commercial or financial relationships that could be construed as a potential conflict of interest.
